# *Enhanced Disease Susceptibility1* Regulates Immune Response in *Lotus japonicus*

**DOI:** 10.3390/ijms26083848

**Published:** 2025-04-18

**Authors:** Mengru Yuan, Qiong Li, Mingchao Huang, Hongdou Huang, Chunyu Sun, Huawu Jiang, Guojiang Wu, Yaping Chen

**Affiliations:** 1 Key Laboratory of South China Agricultural Plant Molecular Analysis and Genetic Improvement, Guangzhou 510650, China; yuan10940@foxmail.com (M.Y.); liqiong21@mails.ucas.ac.cn (Q.L.); huangmc08@163.com (M.H.); hongdou98@outlook.com (H.H.); xiamochanming@sina.com (C.S.); hwjiang@scbg.ac.cn (H.J.); wugj@scbg.ac.cn (G.W.); 2 Guangdong Provincial Key Laboratory of Applied Botany, South China Botanical Garden, Chinese Academy of Sciences, Guangzhou 510650, China; 3 University of Chinese Academy of Sciences, Beijing 100049, China

**Keywords:** EDS1, *Lotus japonicus*, *Ralstonia solanacearum*, disease resistance

## Abstract

*Enhanced disease susceptibility1* (*EDS1*) is a key node in the plant immune signaling network, regulating salicylic acid (SA) levels and other immune responses in *Arabidopsis thaliana*. We previously reported that modulation of SA by *AGD2-like defense response protein 1* (*ALD1*) has been shown to influence the immune response in *Lotus japonicus*, but the role of *LjEDS1* in this species remains unclear. Here, we identified and characterized the *LjEDS1* gene in *L. japonicus*. The LjEDS1 protein contains a lipase-like domain and an EP domain similar to the Arabidopsis EDS1 protein. Subcellular localization studies revealed that the LjEDS1 protein is distributed in both the cytoplasm and nucleus. Heterologous expression of *LjEDS1* in the Arabidopsis *ateds1* mutant increased resistance to *Pseudomonas syringae* pv. *Tomato (Pst)* strain DC3000. In *L*. *japonicus*, roots of the *ljeds1* mutants exhibited heightened susceptibility to *Ralstonia solanacearum*, with increased lesion areas and bacterial titers. Conversely, the overexpression of *LjEDS1* reduced the lesion areas and bacterial titers in roots infected with *R*. *solanacearum* compared to those in the wild-type. Gene expression analysis showed that *LjEDS1* regulates defense-related, basal immunity, and oxidative stress response genes in *L*. *japonicus* roots. These findings establish *LjEDS1* as an important regulator of disease resistance in *L*. *japonicus*.

## 1. Introduction

Plants are continuously exposed to a variety of pathogens that can restrict nutrients, disrupt physiological processes, and cause tissue damage through toxins, cell-wall-degrading enzymes, and virulence proteins. These disruptions result in symptoms such as wilting, decay, excessive growth, dysplasia, and even death [1]. To counter these threats, plants have developed innate immune responses relying on two primary recognition systems. The first detects pathogen-associated molecular patterns (PAMPs) via pattern recognition receptors (PRRs) on the plasma membrane, activating PAMP-triggered immunity (PTI) through mitogen-activated protein kinase (MAPK) cascades. If pathogens secrete effectors that inhibit PTI, plants activate effector-triggered immunity (ETI) through nucleotide-binding leucine-rich repeat (LRR) receptors (NLRs), triggering robust defense responses, including transcriptional reprogramming and developmental adjustments to thwart pathogen invasion [2,3,4].

*Enhanced Disease Susceptibility 1* (*EDS1*) is central to plant immune response activation, coordinating multiple regulatory pathways to maintain immune system functionality when one pathway is disrupted by pathogen effectors. PTI triggered by leucine-rich repeat receptor proteins in Arabidopsis depends on the EDS1-PAD4-activated disease resistance1 (ADR1) complex [5]. Pathogens often target EDS1 by secreting effectors to suppress PTI. In response, plants utilize EDS1 as bait to sense pathogen effectors, activating ETI and protecting EDS1 via resistant protein (R protein), triggering programmed cell death and inducing the expression of resistance-related genes in the nucleus, ultimately inhibiting further infection with pathogens [6,7]. Additionally, systemic acquired resistance (SAR) requires salicylic acid (SA) [8], which is promoted by the EDS1/phytoalexin deficient 4 (PAD4) heterodimer through activation of genes such as *Isochoristmate synthase1* (*ICS1*) and *AGD2-like defense response protein 1* (*ALD1*), leading to SA accumulation [9,10].

*Lotus japonicus* is a model legume species whose roots interact with beneficial microorganisms, and many pathogenic microbes in the soil must be identified and resisted. This dual interaction necessitates precise and efficient defense mechanisms. *Ralstonia solanacearum*, a compatible pathogen of *L. japonicus*, invades root tissues, colonizes xylem vessels, and replicates within the host, ultimately leading to wilting and death [4,11].

The function of *LjEDS1* in the immune response of *L. japonicus* has been poorly understood. Our previous studies found that *LjALD1* regulates SA levels in *L. japonicus*, thereby positively affecting resistance to *R*. *solanacearum* [11,12]. In this study, we identified the *LjEDS1* gene and analyzed its expression patterns in *L. japonicus*. We investigated its role in disease resistance using Arabidopsis *ateds1* mutants and confirmed *LjEDS1*’s positive role in resistance to *R. solanacearum* by analyzing *LjEDS1*-overexpressing and *ljeds1* mutant plants. Our findings elucidate the function of *LjEDS1* in the immunity of *L. japonicus*.

## 2. Results

### 2.1. LjEDS1 Is an EDS1 Orthologs in L. japonicus

The coding sequence (CDS) of the *EDS1* gene in *L*. *japonicus* is 1836 bp and is named *LjEDS1* (Lj1g3v0416380). This sequence is divided into three exons within the genome sequence of 3260 bp and encodes a protein that contains both a lipase-like domain belonging to the Abhydrolase superfamily and the EP (EDS1-PAD4) domain of the EP superfamily. A homologous gene, *LjEDS1-like* (Lj1g3v0416340), designated *LjEDS1L*, was also identified (Supplementary Figure S1). Phylogenetic analysis of EDS1, PAD4, and senescence-associated gene101 (SAG101) in the *EDS1* family showed that LjEDS1 clusters in the same evolutionary branch as EDS1 in *Glycine max* (soybean) and Arabidopsis (Supplementary Figure S2). Protein sequence alignments revealed that LjEDS1 and LjEDS1L possess characteristic EDS1 motifs, including SDH in the lipase-like domain and EPLDIA in the EP domain [13,14,15]. However, LjEDS1L lacks the KNEDT motif, located in the EP domain (Supplementary Figure S3). Consequently, this study focused on the role of *LjEDS1* in disease resistance.

**Figure 1:**
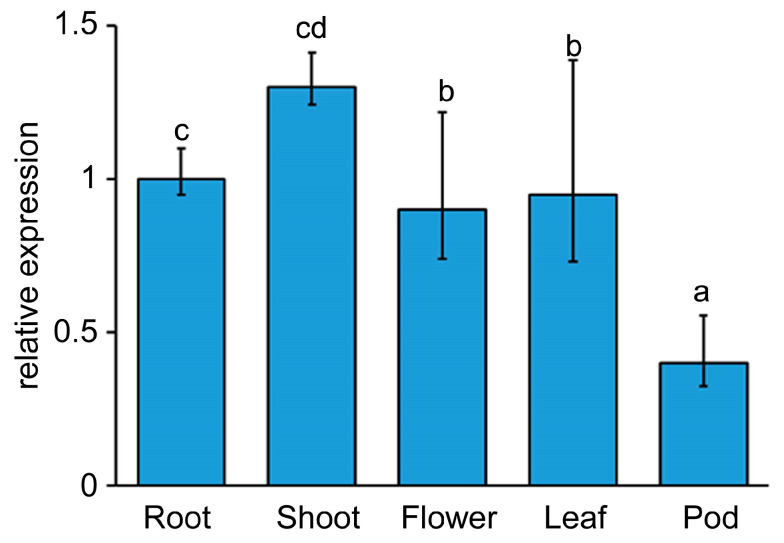
Specific expression pattern analysis of *LjEDS1* in *L. japonicus*. RNA was extracted from 10-day-old roots, shoots, leaves, flowers, and pods of MG-20, and qRT-PCR analysis was performed. The expression level in the root was used as a reference. The data are the mean ± SD of three biological replicates. Lowercase letters above the bars indicate significant differences (one-way ANOVA, *p* < 0.05). Relative expression was normalized to that of the reference gene *LjATPase* (internal control).

**Figure 2:**
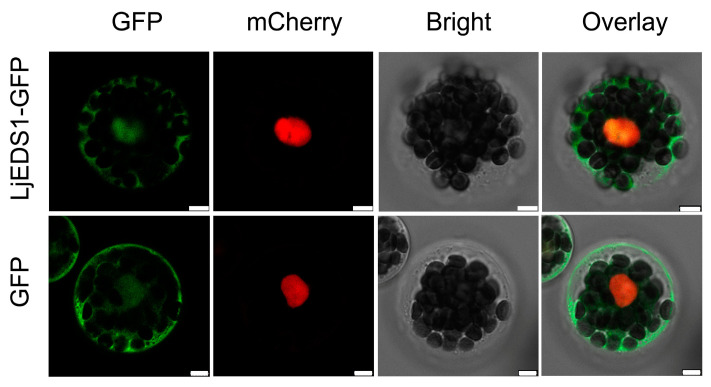
Subcellular localization of LjEDS1 protein. Confocal images of Arabidopsis protoplasts transiently expressing LjEDS1-GFP fusions. Green fluorescence of GFP and LjEDS1-GFP fusions; red fluorescence from mCherry fused with a nuclear localization signal (NLS) peptide; bar = 5 μm.

Tissue-specific expression analysis of *LjEDS1* in *L. japonicus* showed the highest expression levels in shoots, followed by roots, flowers, and leaves, with the lowest expression observed in pods (Figure 1). The LjEDS1 protein was expressed in Arabidopsis mesophyll protoplasts and localized to both the cytoplasm and nucleus (Figure 2).

### 2.2. LjEDS1 Positively Regulates Disease Resistance to Pst DC3000 in Arabidopsis

To investigate the biological roles of the *LjEDS1* gene in plant immunity, *LjEDS1* was overexpressed in the *eds1* null mutant line of *eds1-2* (*ateds1*) in Arabidopsis [13]. Positive transgenic plants were identified using genomic sequence fusion of *LjEDS1* with the β-glucuronidase (GUS) reporter gene to generate p35S::*LjEDS1*- GUS transgenic Arabidopsis lines (Supplementary Figure S4A). Two homozygous transgenic Arabidopsis *eds1-2* lines expressing *LjEDS1* were selected for subsequent studies. GUS signals confirmed their positive transgenic status (Supplementary Figure S4B). Semi-quantitative RT-PCR demonstrated increased expression levels of the *LjEDS1* gene in transgenic lines (Supplementary Figure S4C).

**Figure 3:**
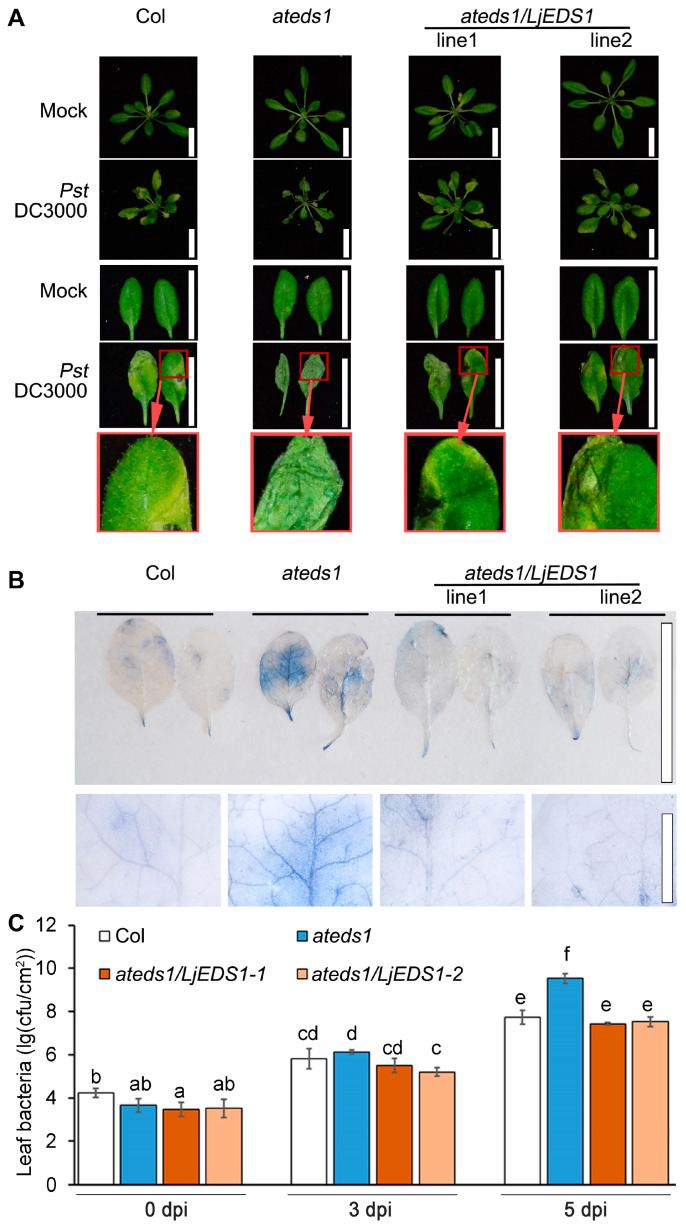
*LjEDS1* restores plant pathogen resistance of the *eds1*-2 mutated allele in Arabidopsis. (**A**) Disease resistance phenotypes of Col, *ateds1*, and two representative *LjEDS1* complementation transgenic lines (*ateds1/LjEDS1*). Four-week-old plants were dip-inoculated with *Pst* DC3000 (OD_600_ = 0.0002). Leaves shown were photographed at 5 days post infection (dpi); bar = 2 cm. (**B**) Cell death analysis of Col, *ateds1*, and *ateds1/LjEDS1* complementation transgenic plants. Stained leaves as described in (**A**) at 5 dpi with trypan blue. Representative leaves were from six plants per genotype. Experiments were independently repeated three times; bar = 1 cm. (**C**) Bacterial growth of *Pst* DC3000 on plants described in (**A**) at 0, 3, and 5 dpi. cfu/cm^2^, colony-forming units per cm^2^ of leaves. Data are the mean ± SD of three biological replicates. Lowercase letters indicate significant differences (one-way ANOVA, *p* < 0.05).

Under normal growth conditions, no morphological differences were observed among Col, *ateds1*, and *ateds1*/*LjEDS1* plants. Leaves from 4-week-old plants (positions 5–9) of wild-type, *ateds1*, and *ateds1/LjEDS1* lines were inoculated with *Pst* DC3000. At 5 days post infection (dpi), *ateds1* leaves exhibited almost complete necrosis and wilting, whereas Col and *ateds1*/*LjEDS1* plants showed significantly reduced lesion areas (Figure 3A). Trypan blue staining confirmed extensive cell death in *ateds1* leaves, which was mitigated in Col and *ateds1*/*LjEDS1* lines (Figure 3B). Additionally, bacterial titers in *ateds1*/*LjEDS1* leaves were 10–20% lower than those in *ateds1* leaves at 5 dpi (Figure 3C). These results indicate that *LjEDS1* can complement the *eds1-2* defect in regards to pathogen resistance.

### 2.3. Differential Gene Expression Analysis in ljeds1 Roots

**Figure 4:**
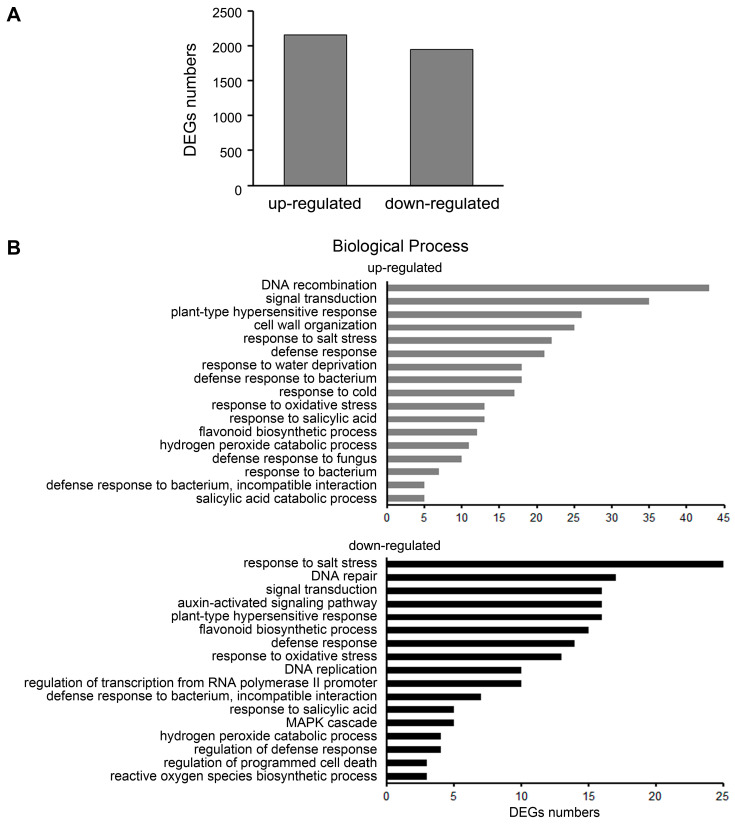
Analysis of differentially expressed genes (DEGs) in Gifu B-129 ecotype and its *ljeds1* mutant of *L. japonicus* (Gifu B-129 ecotype). (**A**) Histogram showing the number of upregulated and downregulated DEGs between *ljeds1* mutant and WT plants. (**B**) GO enrichment analysis of DEGs in biological processes between *ljeds1* mutant and WT plants.

We obtained two *LORE1* insertion mutant lines (plant IDs in *Lotus* Base: 30014411 and 30012794) with a *L*. *japonicus* Gifu B-129 background (https://lotus.au.dk/, accessed on 11 November 2014). Quantitative RT-PCR analysis showed that *LjEDS1* was not expressed in these mutants; thus, these alleles were named *ljeds1-1* and *ljeds1-2* (Supplementary Figures S1 and S5). To investigate the molecular mechanism of *LjEDS1*’s role in immune regulation in *L*. *japonicus*, transcriptome analysis was performed on seedling roots of Gifu B-129 and its mutant *ljeds1-1* (plant IDs in *Lotus* Base: 30014411). A total of 12,801 differentially expressed genes (DEGs) were identified between *ljeds1-1* and Gifu B-129, of which 2160 were upregulated and 1949 were downregulated (Figure 4A). To validate the accuracy of the RNA-seq data, we tested the relative expression levels of seven representative genes involved in plant–microbe interaction by qRT-PCR and found that their expression trends were consistent with the transcriptome data (Supplementary Figure S6). Among the DEGs, the transcription levels of multiple defense response-related genes were significantly downregulated (Supplementary Table S2), including homologous genes predicted by *Lotus* Base for *pathogenesis-related protein1* (Lj0g3v0215119.1), which decreased more than 100-fold. Reactive oxygen species (ROS)-related genes, such as homologous genes of *peroxidase 3-like* (Lj3g3v2890760.1 and Lj3g3v2890770.2), homologous genes of *cationic peroxidase 1-like* (Lj0g3v0012529.1), and homologous genes of *peroxidase 10-like* (Lj2g3v1836090.1), showed a 3–10-fold decrease. Transcription levels of homologous genes (Lj2g3v3246900.1 and Lj2g3v3315570.1) of *red chlorophyll catabolite reductase,* associated with programmed cell death, decreased by more than 10-fold [16].

Gene ontology (GO) enrichment analysis was conducted to determine the primary biological functions of DEGs in *ljeds1*. Upregulated categories included DNA recombination, signal transduction, plant-type hypersensitive response, responses to abiotic stress, defense responses to biotic stress, and responses to SA. Downregulated categories included responses to salt stress, defense responses to biotic stress, DNA repair, signal transduction, ROS synthesis and metabolism, SA synthesis, MAPK cascade, and regulation of programmed cell death (Figure 4B). These findings indicate that *LjEDS1* plays a critical role in regulating the defense response in *L. japonicus*. Furthermore, a Kyoto Encyclopedia of Genes and Genomes (KEGG) enrichment analysis was performed to identify the associated signaling pathways. Significant enrichment was observed in pathways including flavonoid biosynthesis (homologous genes of *chalcone synthase*: Lj2g3v2125830.1, Lj4g3v2574990.1, and Lj2g3v2124310.1; homologous genes of *chalcone isomerase*: Lj5g3v2288880.1, etc.), MAPK signaling pathway (homologous genes of *mitogen-activated protein kinase kinase kinase 1-like*: Lj6g3v2256790.1 and Lj4g3v0335750.1, etc.), and phenylpropanoid biosynthesis (homologous genes of *isoliquiritigenin 2′-O-methyltransferase-like*: Lj0g3v0215539.1 and Lj0g3v0273599.1; homologous genes of *phenylalanine ammonia-lyase*: Lj3g3v0602630.2 and Lj3g3v0602620.1; homologous genes of *peroxidase 3-like* Lj3g3v2890760.1; homologous genes of *cationic peroxidase 1-like*: Lj0g3v0012529.1; homologous genes of *peroxidase 10-like*: Lj2g3v1836090.1, etc.). These results demonstrate the crucial role of *LjEDS1* in signaling pathways such as MAPK and in plant hormones (Supplementary Table S3).

### 2.4. ljeds1 Mutants Are Susceptible to R. solanacearum

**Figure 5:**
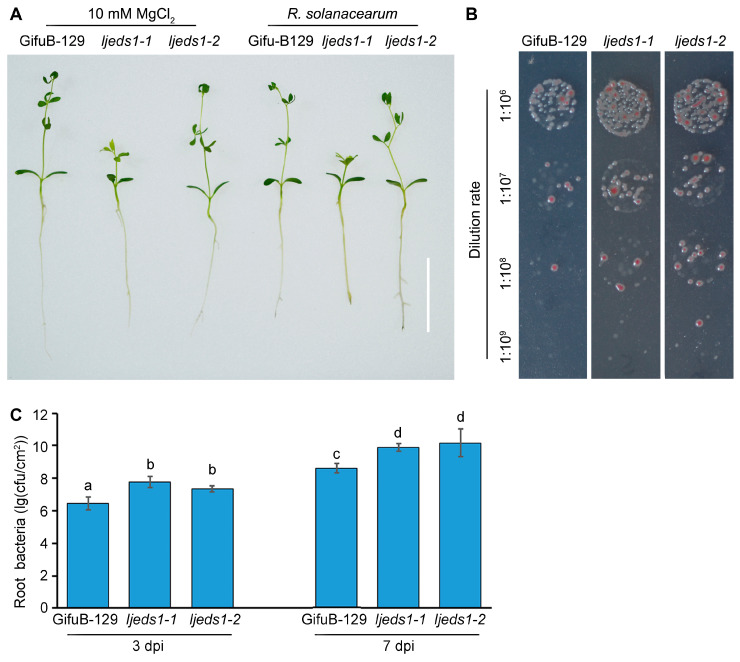
The disease resistance phenotype of *LjEDS1*-related lines under *R*. *solanacearum* infection in *L*. *japonicus* (Gifu B-129 ecotype). (**A**) Three-day-old plants were infected with *R*. *solanacearum* (OD_600_ = 0.01), and roots were photographed at 7 dpi; bar = 2 cm. (**B**) Colonization of *R*. *solanacearum* in roots at 7 dpi on SMSA medium. (**C**) Bacterial growth of *R*. *solanacearum* in *LjEDS1*-related lines at 3 dpi and 7 dpi. cfu/cm, colony forming units per cm of roots. Data are the mean ± SD of three biological replicates. Lowercase letters indicate significant differences (one-way ANOVA, *p* < 0.05).

To evaluate the role of *LjEDS1* in the immune response of *L*. *japonicus*, the disease resistance phenotype of Gifu B-129 and *ljeds1* mutants (*ljeds1-1* and *ljeds1-2*) was assessed. Roots of 3-day-old seedlings were inoculated with *R*. *solanacearum*, and observations were recorded at 3 dpi and 7 dpi. At 7 dpi, browning lesions were more pronounced in *ljeds1-1* and *ljeds1-2* compared to in Gifu B-129 (Figure 5A). Root bacterial titers were significantly higher in *ljeds1* mutants, with titers approximately 10-fold higher at 3 dpi and 20-fold higher at 7 dpi compared to those for Gifu B-129 (Figure 5B,C). These results indicate that the *ljeds1* mutation accelerates *R. solanacearum* proliferation in the Gifu B-129 ecotype.

### 2.5. LjEDS1 Overexpression Enhances Resistance to R. solanacearum

**Figure 6:**
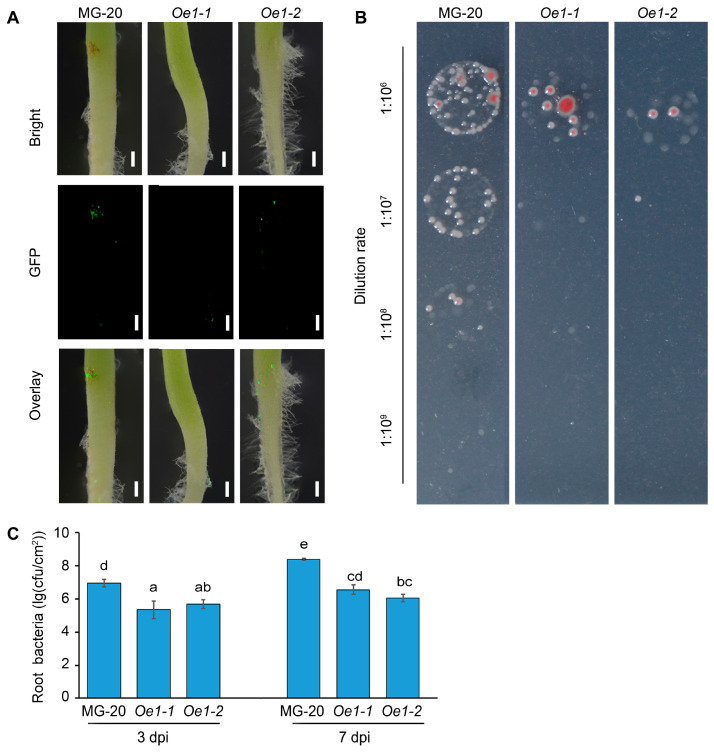
The disease resistance phenotype of *LjEDS1* overexpression lines under *R*. *solanacearum* infection in *L*. *japonicus* (MG-20 ecotype). (**A**) Three-day-old plants were infected with *R*. *solanacearum*-GFP (OD_600_ = 0.01), and roots were photographed at 7 dpi; bar = 1 mm. (**B**) Colonization of *R*. *solanacearum* in roots at 7 dpi on SMSA medium. (**C**) Bacterial growth of *R*. *solanacearum* in *LjEDS1*-related lines at 3 dpi and 7 dpi. cfu/cm, colony-forming units per cm of roots. Data are the mean ± SD of three biological replicates. Lowercase letters indicate significant differences (one-way ANOVA, *p* < 0.05).

To further confirm the role of *LjEDS1* in the immune response of *L*. *japonicus*, two *LjEDS1* overexpression (Oe) lines (*Oe1-1* and *Oe1-2*) were generated in the MG-20 ecotype. Quantitative RT-PCR analysis showed high *LjEDS1* expression in Oe lines (Supplementary Figure S7C). Roots of 3-day-old seedlings were inoculated with *R. solanacearum-GFP*, and observations were made at 3 dpi and 7 dpi. At 7 dpi, brown lesions were observed on MG-20 roots but were absent in the Oe1-1 and Oe1-2 lines. Fluorescence microscopy revealed reduced *R. solanacearum* aggregation in the Oe lines compared to the results for MG-20 (Figure 6A). Statistical analysis showed that bacterial titers in the Oe lines were reduced by approximately 100-fold at both 3 dpi and 7 dpi compared to those for MG-20 (Figure 6B,C). These results indicate that the expression level of *LjEDS1* positively correlates with the defense response of *L. japonicus* to *R. solanacearum*.

## 3. Discussion

Studies on Arabidopsis, soybean, and tobacco have shown that EDS1 contains two domains: the lipase-like domain of the abhydrolase superfamily and the EP domain of the EP superfamily. Among these, the EP domain is critical for the immune function of EDS1 protein family members [14,17,18,19,20]. Domain prediction analysis revealed that the LjEDS1 protein sequence contains both domains (Supplementary Figure S3). Additionally, it possesses the KNEDT motif, which is unique to EDS1 compared to the composition of other family members such as SAG101 and PAD4. This motif indicates that LjEDS1 is likely a homolog of AtEDS1 [13,14,15]. The conserved structure of EDS1 across evolution is further supported by the evolutionary relationship of EDS1 family members in *L*. *japonicus*, Arabidopsis, and soybean. The phylogenetic analysis shows that LjEDS1 clusters within the EDS1 clade, forming an independent branch distinct from SAG101 and PAD4 (Supplementary Figure S2).

Previous research on EDS1 has primarily focused on its function in the leaves. For instance, AtEDS1 collaborates with immune partners like PAD4 to regulate immune responses in Arabidopsis leaves via SA-dependent and SA-independent pathways [21]. OsEDS1 regulates pathogen susceptibility in rice leaves through the jasmonic acid signaling pathway [22], while GmEDS1 modulates SA accumulation, enhancing soybean leaf sensitivity to virulent pathogens [17]. In contrast, *LjEDS1* exhibits a unique expression pattern, with higher expression levels in shoots and roots compared to those in flowers, leaves, and pods (Figure 1). Stem-cell-triggered immunity safeguards plant shoot apexes from pathogen infection [23], suggesting that this expression pattern may implicate *LjEDS1* in a unique immune mechanism in shoot or root apexes. Meanwhile, its high expression in roots supports a flexible and efficient immune response when roots interact with the complex, microbe-rich soil environment. The subcellular localization of EDS1 between the nucleus and cytoplasm is essential for its function. The EDS1-SAG101 heterodimer predominantly localizes to the nucleus, while the EDS1-PAD4 heterodimer is found in both the cytoplasm and nucleus. The SAG101-EDS1-PAD4 ternary complex resides in the nucleus [24]. SAG101 promotes the nuclear entry of EDS1, while PAD4 maintains a cytoplasmic–nuclear balance [25,26]. LjEDS1 localization to both the cytoplasm and nucleus (Figure 2) suggests that its regulatory mechanism in *L. japonicus* may be similar to that of EDS1 in other plants, such as Arabidopsis, where cytoplasmic and nuclear shuttling enables immune regulation.

To preliminarily assess the immune function of *LjEDS1*, we conducted heterologous complementation experiments. Expression of *LjEDS1* in the Arabidopsis *ateds1* mutant restored pathogen resistance, reducing lesion formation and bacterial titers to wild-type levels (Figure 3). This finding is consistent with studies on *GmEDS1*, which partially compensates for pathogen resistance defects in Arabidopsis *ateds1* mutants [17]. These results suggest that *LjEDS1* shares structural and functional similarities with known EDS1 proteins and plays a significant role in the immune pathways of *L. japonicus*, particularly in the roots, which are critical for legumes.

Despite substantial studies on *EDS1* in leaves, its role in roots remains underexplored. Specific expression analysis revealed that *LjEDS1* exhibits relatively high expression in the roots of *L. japonicus* (Figure 1). Roots employ cooperative or defensive strategies when encountering foreign microbes. What role does *LjEDS1* play in this defense process? Our study shows that although the targeted tissues differ, *EDS1* performs similar functions within *L. japonicus* roots. Suppression of *EDS1* expression during pathogen infection results in more pronounced necrotic lesions and increased bacterial titers in Arabidopsis, wilting and chlorosis in cotton seedlings, and heightened chlorosis and cell death in soybeans [17,27,28]. In *L*. *japonicus*, *ljeds1* mutants exhibited accelerated pathogen proliferation and larger root lesions upon *R*. *solanacearum* infection compared to the results for wild-type plants (Figure 5). Conversely, overexpression of *LjEDS1* reduced pathogen proliferation and alleviated disease symptoms (Figure 6). These results indicate that *LjEDS1* positively regulates defense responses in *L*. *japonicus* roots.

After confirming the regulatory role of EDS1 in the immune function of *L. japonicus*, we investigated its underlying regulatory mechanisms. GO enrichment analysis indicated that the mutation of *ljeds1* affected several mechanisms related to defense responses (Figure 4B), highlighting the transcriptional regulatory role of *LjEDS1* in immune function. Previous studies on Arabidopsis have shown that excessive accumulation of EDS1 in the nucleus can trigger autoimmunity, leading to symptoms resembling pathogen infection, such as inhibited root growth, increased root hair formation, and programmed cell death [29,30,31]. Additionally, the EDS1-SAG101 complex interacts with NRG1 (N requirement gene 1) to induce host cell death. GO analysis revealed that the *ljeds1* mutation affected DEGs related to programmed cell death and plant-type hypersensitive responses (Figure 4B). This suggests that the significantly shorter root length observed in *LjEDS1*-overexpressing lines might result from autoimmunity triggered by *LjEDS1* (Supplementary Figure S5A). Collectively, these results demonstrate that *LjEDS1* regulates immune responses in *L. japonicus*, including root growth inhibition and programmed cell death.

Plant physiological responses require various signaling molecules. One of the earliest markers of plant immune responses is the production of ROS in different subcellular compartments [32]. In this study, biosynthesis and metabolic processes related to ROS were altered in *ljeds1* mutants (Figure 4B). Under conditions such as pathogen infection or cold stress, EDS1 regulates ROS production and scavenging through interactions with proteins like PAD4 and SAG101, thereby modulating plant resistance and immune responses [5,33]. SA maintains the ROS balance upstream [32]. Additionally, the SA-induced EDS1-NPR1 complex binds to promoters of defense genes, activating their roles in Arabidopsis immunity [34]. EDS1 enhances resistance to biotic stress by regulating SA accumulation [13]. In *Chrysanthemum morifolium*, transcriptional regulation mediated by *CmEDS1* plays a vital role in early-stage infection before lesion formation, supporting SA accumulation for defense [35]. Similarly, in cotton, *GbEDS1* enhances resistance by regulating SA and H_2_O_2_ production during disease development [27]. HIR3 in tobacco also promotes resistance through an EDS1- and SA-dependent pathway, causing cell death and SA accumulation [36]. Throughout the defense process, AtEDS1 forms a complex with DELLA protein, negatively regulated by gibberellin (GA), which interacts with SA to balance growth and defense [28,37]. In this study, GO enrichment analysis revealed that mechanisms related to the SA catabolic process and response to SA were regulated by *LjEDS1* (Figure 4B). These results align with those from previous studies on *EDS1*, suggesting that *LjEDS1* modulates plant defense by regulating SA and ROS accumulation.

Additionally, GO and KEGG enrichment analysis revealed that the MAPK cascade was downregulated in *L. japonicus* upon *ljeds1* mutation (Figure 4B). MAPKs are crucial in plant immune responses [10,38]. MAPKs can bypass SA to induce SA-responsive genes and act upstream of ROS bursts [39,40]. In Arabidopsis, MAP kinase 4 (MPK4) regulates SA-dependent responses through EDS1 and PAD4. Our findings suggest that *LjEDS1* may influence MAPK pathways in *L. japonicus*. While *LjEDS1* modulates immune responses in *L. japonicus* roots through a complex regulatory network, further investigation is required to elucidate its specific regulatory mechanisms.

**Figure 7:**
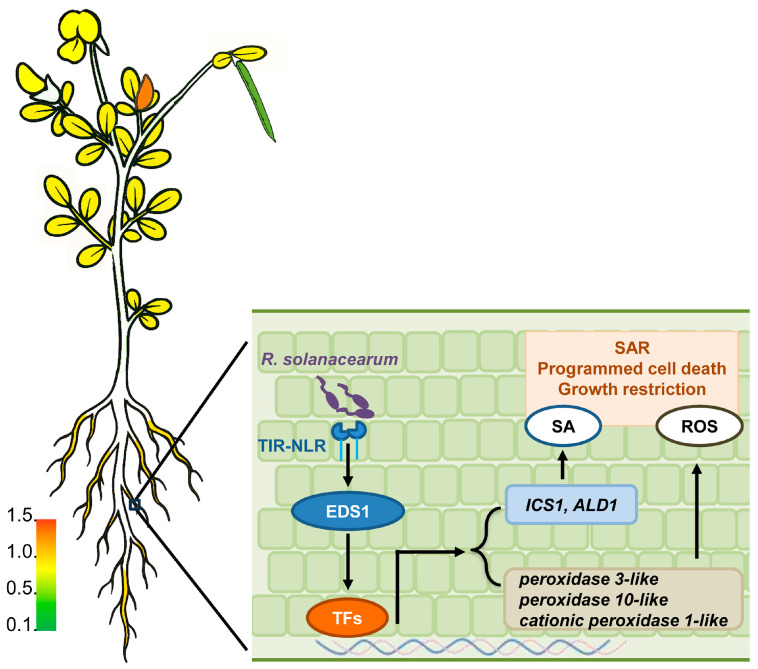
Gene expression model of the response of *L*. *japonicus* to *R*. *solanacearum* infection. The left image shows the tissue-specific expression pattern of *LjEDS1* genes. The right image shows the working model for *LjEDS1* immune signaling in *L*. *japonicus*.

In conclusion, during *R. solanacearum* infection of *L. japonicus* roots, the plant’s NLRs detect pathogen effectors, thereby activating *EDS1*. The active *EDS1* triggers downstream transcription factors (TFs), which subsequently induce the transcription of defense-related genes (Figure 7). These include the SA biosynthesis gene *ICS1* and *ALD1* oxidation-reduction reaction related genes such as *peroxidase 3*-like, which trigger pathogen resistance.

## 4. Materials and Methods

### 4.1. Growth Conditions and Treatments of L. japonicus

Two ecotypes of *L. japonicus*, Gifu B-129 and MG-20, were primarily used in this study. *LORE1* insertion mutant lines of *ljeds1-1* and *ljeds1-2* (plant IDs in *Lotus* Base: 30014411 and 30012794) with a Gifu B-129 background were obtained from *Lotus* Base (https://lotus.au.dk/, accessed on 11 November 2014). Two *LjEDS1* overexpression lines (*Oe1-1* and *Oe1-2*) were generated by transforming MG-20 with *Agrobacterium tumefaciens* strain AGL1. The *L. japonicus* ecotypes Gifu B-129 and MG-20 were used as wild-type (WT) controls in this study. After surface disinfection, the seeds were placed in sterile dishes for 2 days and grown in a growth chamber under a long-day photoperiod (16 h light/8 h dark) at 22 ± 2 °C.

For bacterial growth assays, *R*. *solanacearum* was cultured in SMSA medium at 28 °C in the dark, and 2-day-old seedlings were infected by adding *R*. *solanacearum* (suspended in sterile 10 mM MgCl_2_ to OD_600_ = 0.01, equivalent to ~2 × 10^6^ cfu mL⁻^1^) or a mock suspension (10 mM MgCl_2_). The images were observed under a Leica M165 FC Fluorescent Stereo Microscope. The root tips (1 cm) were excised at specific points in time post-inoculation, ground, and cultured on SMSA solid medium for 2 days. Data were statistically analyzed [11]. The experiments were repeated three times, with three plants per sample.

### 4.2. Growth Conditions and Treatments of Arabidopsis

The Arabidopsis ecotype Columbia-0 (Col-0) was used as the wild-type (WT) in this study. After surface disinfection, the seeds were incubated for 2 days in the dark at 4 °C and then grown in a growth chamber under a short-day photoperiod (12 h light/12 h dark) at 22 ± 2 °C.

For bacterial growth assays, the *Pseudomonas syringae* pv. *Tomato* DC3000 was cultured in King’s B medium containing 25 mg mL⁻^1^ rifampicin at 28 °C. Leaves of 4-week-old plants were dip-inoculated with *Pst* DC3000 (suspended in sterile 10 mM MgCl_2_ to OD_600_ = 0.0002, equivalent to ~10^5^ cfu mL⁻^1^) or a mock suspension (10 mM MgCl_2_). Samples were collected at 0, 3, and 5 days post infection (dpi). Each sample included three independent biological replicates, with three infected leaf discs per replicate. Leaf discs were placed in microcentrifuge tubes, ground with a pestle in 1 mL of 10 mM MgCl_2_, then diluted, cultured on King’s B solid medium, and statistically analyzed [41].

### 4.3. RNA-Seq and Transcriptome Data Analysis

Root samples from 10-day-old seedlings were collected and sent to Shanghai OE Biotech Co., Ltd. (Shanghai, China) for RNA sequencing. The expression abundance of each gene across the samples was determined through sequence similarity alignment against a database comprising known reference genome sequences and annotation files, using HTSeq count software (Version number: 0.6.0) to obtain the number of reads aligned to genes in each sample and Cufflinks software (Version number: 2.2.1) to calculate the FPKM (fragments per kilobase per million reads) value of gene expression. Sequencing data were mapped to the genome sequence using Hisat 2 software (Version number: 2.0.5). The calculation of gene expression levels was performed using the FPKM method [42]. According to DESeq software (Version number: 1.18.0), the negative binomial distribution test was used to identify differentially expressed genes (DEGs). Only those unigenes with a *p*-value ≤ 0.05 and a fold change ≥ 2 were designated as differentially expressed. The DEGs were subjected to GO enrichment and KEGG enrichment analyses.

### 4.4. RNA Isolation and Expression Analysis

For expression analysis, the tissues were collected and immediately placed in liquid nitrogen or stored at −80 °C. RNA was extracted using the Plant RNA Kit (Magen R4151-02, Guangdong, China). First-strand cDNAs were synthesized using the GoScript™ Reverse Transcription System (Promega, Madison, WI, USA), according to the manufacturer’s instructions. *AtActin*, *LjActin*, or *LjATPase* were used as internal controls for RT-PCR analysis. All qRT-PCR experiments were performed on a LightCycler^®^ 480 Real-Time PCR System (Mannheim, Germany) under the following amplification conditions: 10 min at 95 °C, followed by 40 cycles of 95 °C for 5 s, 60 °C for 20 s, and 72 °C for 20 s. *LjATPase* was used as the internal control for normalization, and the 2^−△△Ct^ method was applied for real-time RT-PCR analysis [11]. The primers used are listed in Supplementary Table S1.

### 4.5. Plasmid Construction and Plant Transformation

To generate *LjEDS1* (Lj1g3v0416380) overexpression lines in *L*. *japonicus* and Arabidopsis, the *LjEDS1* coding sequence (1836 bp) was amplified by PCR and used to construct pUBI::*LjEDS1*-3×FLAG and p35S::*LjEDS1*-GUS vectors. The primers used are listed in Supplementary Table S1. The expression vector pUBI:*:LjEDS1*-3×FLAG was transformed into *A. tumefaciens* strain AGL1 and used to infect *L*. *japonicus* [43]. The p35S::*LjEDS1*-GUS vector was transformed into *A. tumefaciens* strain GV3101 and used to infect Arabidopsis [44]. Positive transformants were identified by GUS staining and RT-PCR.

### 4.6. Subcellular Localization

To confirm the localization of the LjEDS1 protein, the *LjEDS1* coding region was cloned into a vector containing an N-terminal EGFP fusion to generate 35S::*LjEDS1*-nEGFP. The primers used are listed in Supplementary Table S1. A nuclear marker was generated by fusing mCherry to a nuclear localization signal (NLS) peptide. These plasmids were co-transformed into Arabidopsis mesophyll protoplasts [45]. Fluorescence signals were observed using a Leica TCS SP8 confocal laser scanning microscope (Leica, Wetzlar, Germany).

### 4.7. Histochemistry Analysis

Leaves of p35S::*LjEDS1*-GUS Arabidopsis transgenic lines were stained with GUS staining buffer (1 mM X-Gluc, 0.5 mM K_3_[Fe(CN)_6_], 0.5 mM K_4_[Fe(CN)_6_], 1 mM EDTA, pH 8.0, and 0.1 mM PBS buffer, pH 7.0) at 37 °C for 2–4 h following vacuum infiltration for 15 min. Stained tissues were stored in a decolorizing solution (70% ethanol) for observation [11].

To observe dead cells in Arabidopsis leaves, inoculated leaves were immersed in trypan blue solution (30 mL lactic acid, 30 mL glycerol, 30 g phenol, and 900 mg trypan blue) and gently shaken for 40 min. Stained tissues were destained overnight in chloral hydrate solution [46]. Images were captured using a Leica M165 C Stereo Microscope (Leica, Wetzlar, Germany).

### 4.8. Sequence Analysis and Phylogenetic Tree Construction

Relevant nucleotide and protein sequences were obtained from *Lotus* Base (https://lotus.au.dk/, accessed on 1 September 2014), NCBI (https://www.ncbi.nlm.nih.gov/, accessed on 20 March 2023), and Phytozome (https://jgi.doe.gov/, accessed on 20 March 2023). Analysis of gene structure was conducted using GSDS 2.0 (http://gsds.gao-lab.org/, accessed on 12 March 2018). A phylogenetic tree was constructed using MEGA 6.0 with the neighbor-joining (NJ) method and 1000 bootstrap replicates. Multiple sequence alignments of protein sequences across different plant species were conducted using the T-Coffee Server (https://tcoffee.crg.eu/, accessed on 25 March 2023).

### 4.9. Statistical Analysis

All experiments included three biological replicates, and data are presented as means ± SDs (standard deviations). Statistical significance was assessed using SPSS software (version 21.0). One-way ANOVA, followed by Duncan’s post hoc test, were applied to identify significant differences, with *p* < 0.05 indicated by different letters above bars.

## 5. Conclusions

Our findings reveal that *LjEDS1*’s structure closely resembles that of EDS1 in Arabidopsis and other plants, with localization in both the nucleus and cytoplasm. Additionally, *LjEDS1* enhances disease resistance in the *ateds1* mutant of Arabidopsis. The mutation of *LjEDS1* in *L. japonicus* exacerbates the symptoms of *R. solanacearum* infection, while overexpression mitigates these symptoms. *LjEDS1* regulates the disease-resistance response in *L. japonicus* by modulating the transcription levels of genes associated with biotic (and abiotic) stress responses, ROS and SA synthesis, and the MAPK cascade.

## Data Availability

Data are contained within the article and Supplementary Materials. The raw transcriptome sequencing data generated in this study are not publicly available, but are available from the corresponding author upon reasonable request.

## Supplementary Materials

The following supporting information can be downloaded at: https://www.mdpi.com/article/10.3390/ijms26083848/s1.
