# Supplementary material for: Enhanced Disease Susceptibility1 Regulates Immune Response in Lotus japonicus
Source: Int J Mol Sci. 2025 Apr 18;26(8):3848. doi: 10.3390/ijms26083848 (PMC12027765; doi:10.3390/ijms26083848)
Supplement: Supplementary file 1 [file ijms-26-03848-s001.zip › Supplementary Figures.pdf]

Supplementary Figures

Supplementary Figure S1. The genetic structures of *LjEDS1* and *LjEDS1-like*.

Supplementary Figure S2. Phylogenetic analysis of EDS1 protein in *L. japonicus*, *G. max*, and *A. thaliana*.

Supplementary Figure S3. Homology structure domain alignment of EDS1 proteins.

Supplementary Figure S4. Identification of transgenic lines *ateds1/LjEDS1.*

Supplementary Figure S5. Expression identification of *ljeds1* lines with RT-PCR (GifuB-129 ecotype).

Supplementary Figure S6. Relative changes in gene expression measured by qRT-PCR (GifuB-129 ecotype)

Supplementary Figure S7. Identification of *LjEDS1* overexpression (Oe) lines (MG-20 ecotype).

Supplementary Figure S1

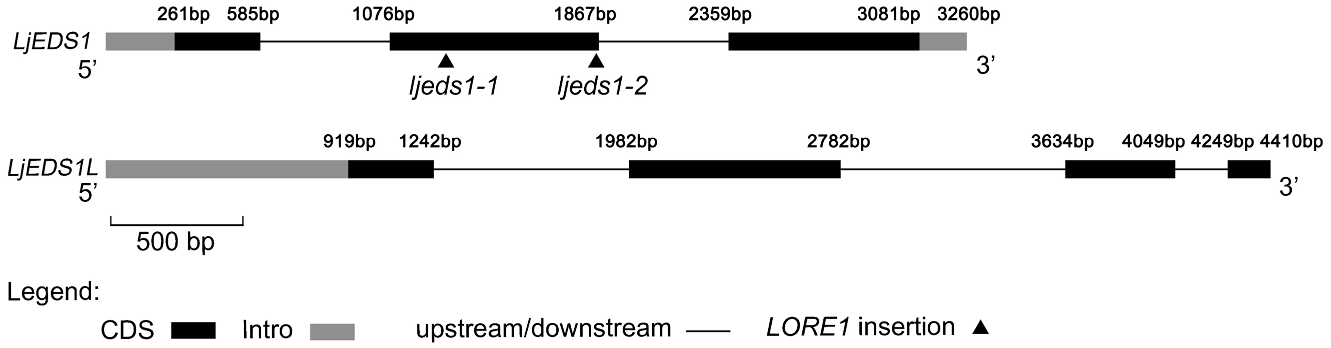

Supplementary Figure S1. The genetic structures of *LjEDS1* and *LjEDS1-like*.

Supplementary Figure S2

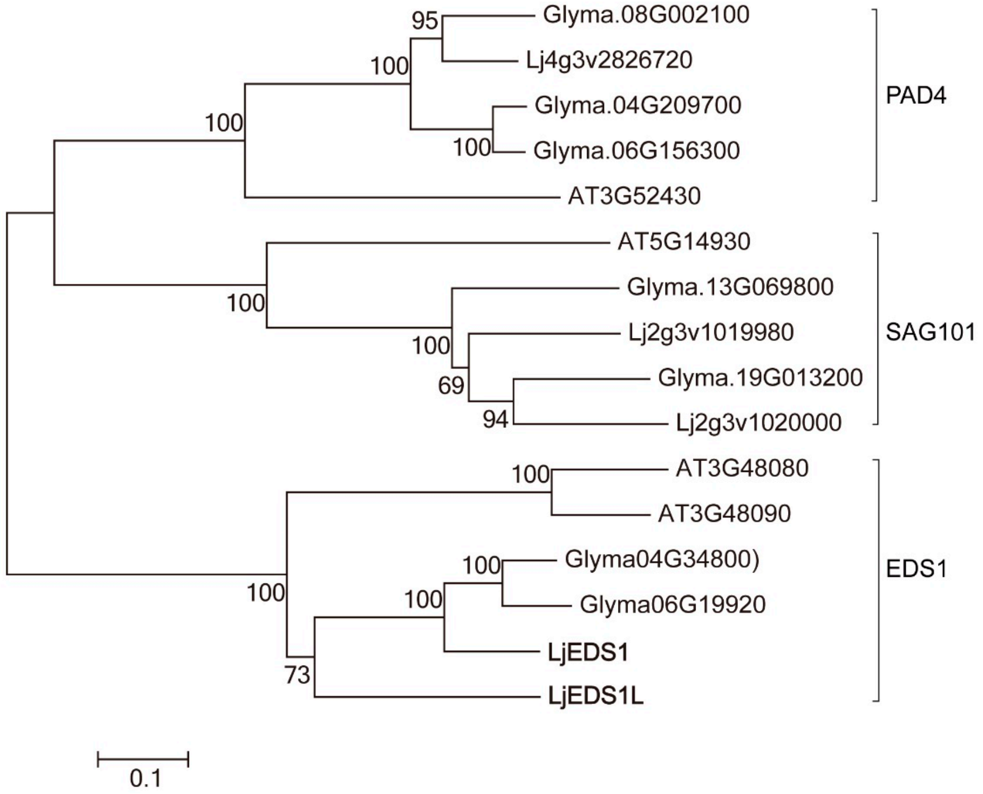

Supplementary Figure S2. Phylogenetic analysis of EDS1 protein in *L. japonicus*, *G. max*, and *A. thaliana*.

Supplementary Figure S3

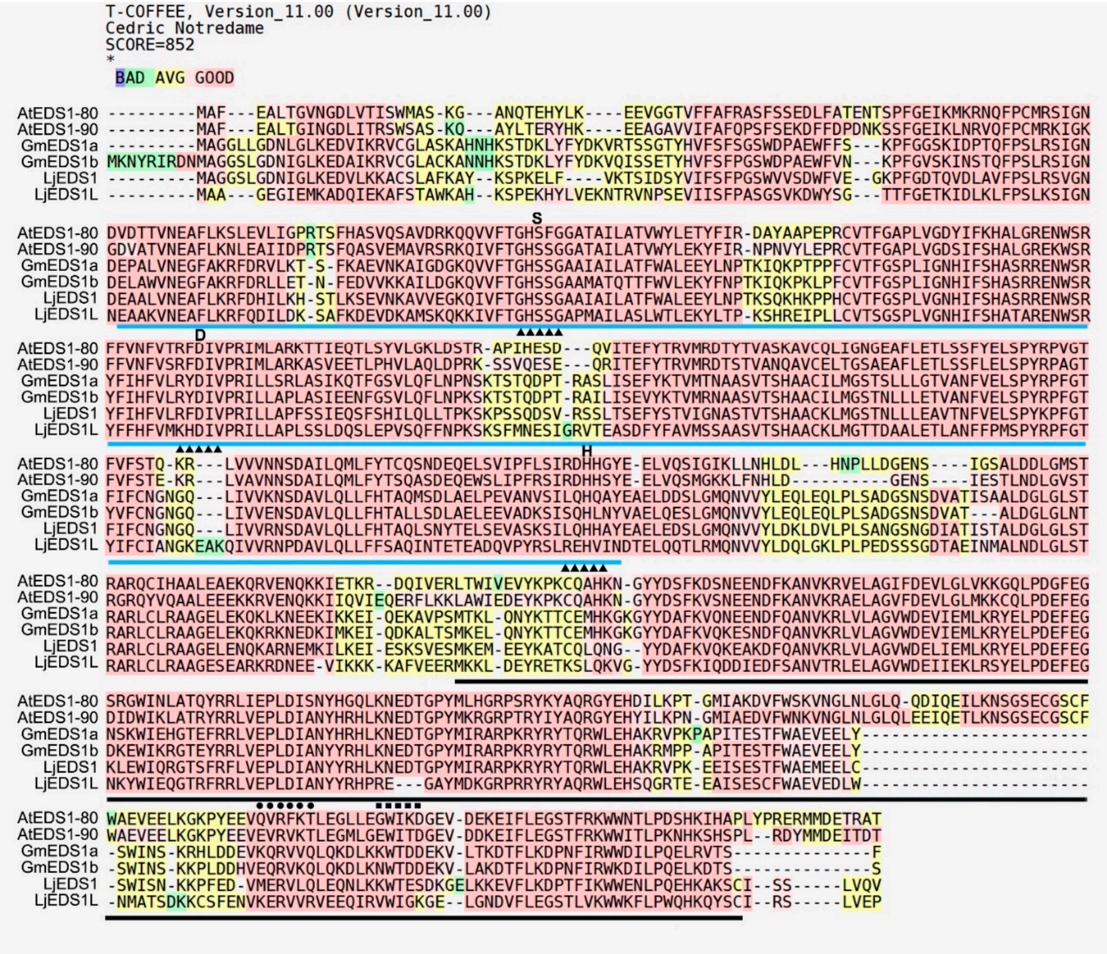

Supplementary Figure S3. Homology structure domain alignment of EDS1 proteins. T-Coffee Server was used for EDS1 protein multi-sequence alignment. Blue lines indicate the lipase-like domain, black lines indicate the EP domain, black triangles indicate the SDH motif, black dots indicate the EPLDIA motif, and black squares indicate the KNEDT motif.

Supplementary Figure S4

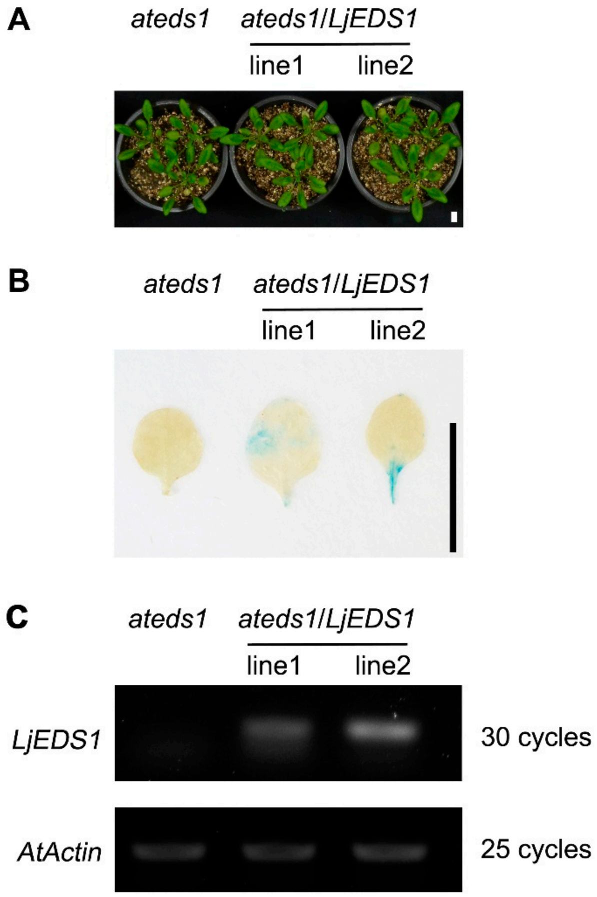

Supplementary Figure S4. Identification of transgenic lines *ateds1*/*LjEDS1.* (A) 4-week-old plants of *ateds1*, and two representative *LjEDS1* complementation transgenic lines *ateds1*/*LjEDS1*. (B) GUS staining showing the expression of *LjEDS1* in Arabidopsis. Bar=1 cm. (C) RT-PCR analysis of *LjEDS1* expression in *ateds1*, *ateds1*/*LjEDS1*. Reference gene *AtActin* amplified with 25 cycles; *LjEDS1* amplified with 30 cycles.

Supplementary Figure S5

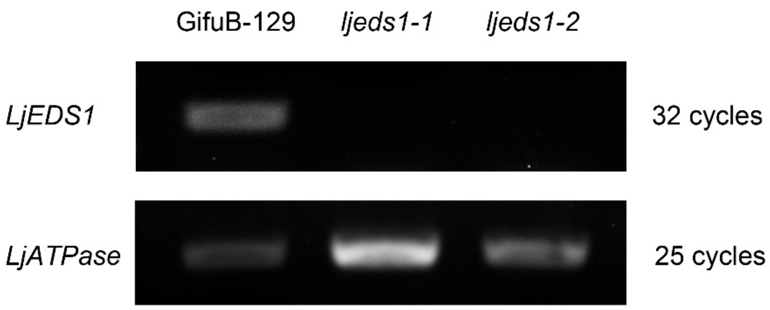

Supplementary Figure S5. Expression identification of *ljeds1* lines with RT-PCR (Gifu B-129 ecotype). There were 25 cycles of the reference gene *LjActin* and 32 cycles of *LjEDS1* amplification.

Supplementary Figure S6

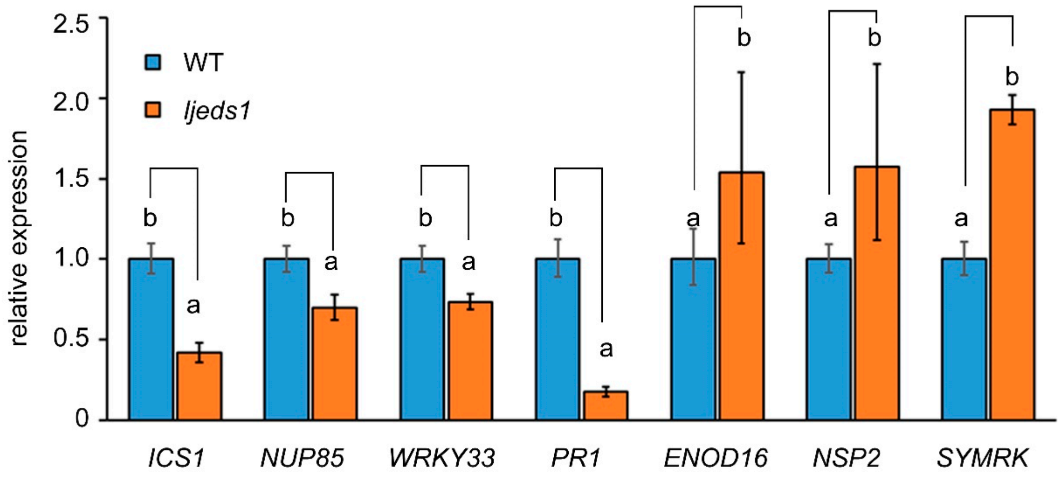

Supplementary Figure S6. Relative changes in gene expression measured by qRT-PCR. Levels of transcript of *ICS1* (Lj3g3v1970100), *NUP85* (Lj1g3v0318210), *WRKY33* (Lj0g3v0238279), *PR1* (Lj0g3v0215119), *ENOD16* (Lj2g3v1728960), *NSP2* (Lj1g3v0785930), *SYMRK* (Lj2g3v1467920) in *L. japonicus*. RNA was extracted from 10-day-old roots, and qRT-PCR analysis was performed. The expression level in the root was used as a reference. The data are the mean ± SD of three biological replicates. Lowercase letters above the bars indicate significant differences (one-way ANOVA, $P < 0.05$). Relative expression was normalized to that of the reference gene *LjATPase* (internal control).

Supplementary Figure S7

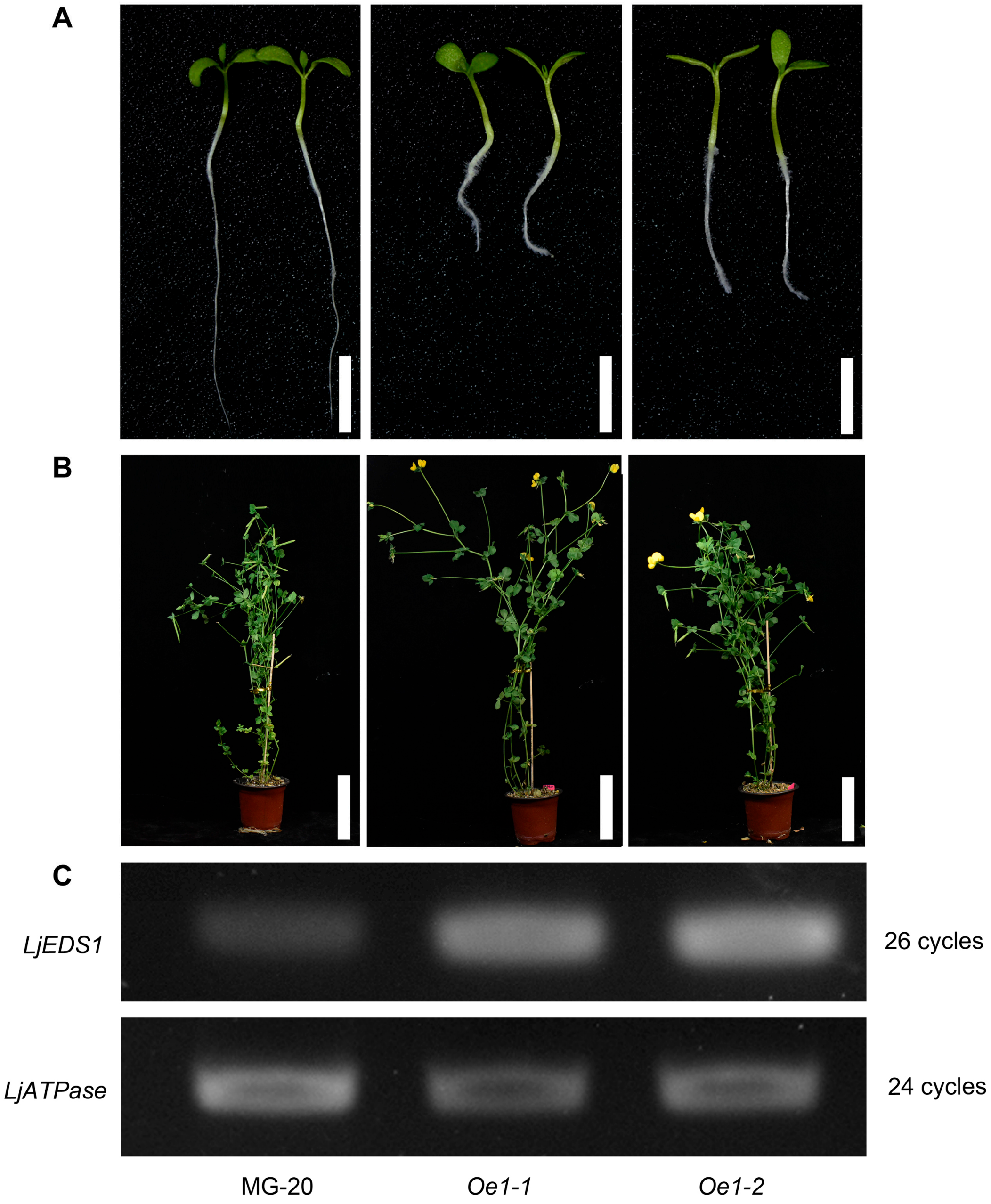

Supplementary Figure S7. Identification of *LjEDS1* overexpression (Oe) lines (MG-20 ecotype). (A) 10-day-old plants of WT and *Oe1* bar=1 cm. (B) 10-week-old plants of WT and *Oe1*, bar=10 cm. (C) Expression identification of *Oe1* lines with RT-PCR. There were 24 cycles of the reference gene *LjATPase* and 26 cycles of *LjEDS1* amplification.
